# Supplementary material for: Reducing risk behaviours after stroke: An overview of reviews interrogating primary study data using the Theoretical Domains Framework
Source: PLoS One. 2024 Apr 26;19(4):e0302364. doi: 10.1371/journal.pone.0302364 (PMC11051587; doi:10.1371/journal.pone.0302364)
Supplement: S1 Table — ✔ denotes domains identified in primary studies included in current study. (DOCX) [file pone.0302364.s001.docx]

**Additional file**

**Theoretical Domains Framework**

Adapted from: Cane J, O’Connor D, Michie S. Validation of the theoretical domains framework for use in behaviour change and implementation research. Implementation Science. 2012;7(1):37.

|  | **TDF Domain** | **Stroke context of theoretical constructs** |
| --- | --- | --- |
| **1**  **🗸** | **Knowledge** (awareness of the existence of something) | Knowledge of nature of stroke, risk factors associated and how to address consequences |
| **2** | **Skills** (ability or proficiency acquired through practice) | Acquire skills to complement knowledge and master through practice the secondary prevention healthy behaviours |
| **3** | **Social/Professional** (role & identity) | Addresses the influence of one’s social role on health behaviours |
| **4**  **🗸** | **Beliefs about capabilities** (acceptance of truth, reality, validity of ability, talent, can put to constructive use) | Encompasses individual self-confidence and perceived ability to perform health behaviours |
| **5** | **Optimism** (confidence things will happen for the best, desired goals attained) | Reflecting the negative versus positive health messaging |
| **6**  **🗸** | **Beliefs about consequences** (acceptance of truth, reality, validity of outcomes of behaviour) | Encompasses the perceptions and expectations of positive or negative outcomes associated with healthy behaviours |
| **7** | **Reinforcement** (increasing probability of response by arranging dependent relationship, or contingency, between response/stimulus) | Acknowledges the external factors associated with healthy behaviours |
| **8**  **🗸** | **Intentions** (conscious decision to perform behaviour/resolve act) | Recognises the personal and conscious decision to perform a behaviour |
| **9** | **Goals** (end states individual wants to achieve) | Addresses goal setting and action planning to achieve healthy behaviour and outcomes |
| **10** | **Memory**, **Attention**, **Decision** **Process** (retain information, focus, choose between alternatives) | Accounts for the cognitive factors that can affect the adoption of healthy behaviours |
| **11** | **Environmental** **context and Resources** (discourages/ encourages development of skills/abilities, independence, social competence, adaptive behaviour) | Encompasses the structured assistance and supports to support healthy behaviours |
| **12** | **Social influences** (interpersonal processes that cause change of thoughts, feelings, behaviours) | Acknowledges the impact of others on individuals’ adopting healthy behaviours |
| **13**  **🗸** | **Emotion** (complex reactions, experiential, behavioural, physiological, in attempting to deal with personally significant event) | Recognises the role emotional reactions after stroke play in influencing healthy behaviours |
| **14** | **Behavioural regulation** (aimed at managing/changing actions – objectively observed/measured) | Ability to self-regulate and control their health behaviours |

Legend: **🗸** denotes domains identified in primary studies included in current study
