# Supplementary material for: Reducing risk behaviours after stroke: An overview of reviews interrogating primary study data using the Theoretical Domains Framework
Source: PLoS One. 2024 Apr 26;19(4):e0302364. doi: 10.1371/journal.pone.0302364 (PMC11051587; doi:10.1371/journal.pone.0302364)
Supplement: S3 Table — (DOCX) [file pone.0302364.s003.docx]

**S3 Table. Template for Intervention Description and Replication (TIDieR) Checklist**

| **RCT** | **1. brief name** | **2. rational; theory; techniques** | **3. what materials** | **4. what procedures** | **5. who provided** | **6. how** | **7. where** | **8. when & how much** | **9. tailoring** | **10. modifications** | **11. how well - planned** | **12. how well - actual** |
| --- | --- | --- | --- | --- | --- | --- | --- | --- | --- | --- | --- | --- |
| **Eames**  2013 | Stroke education & support | Health belief model | online package, tailored written information booklet, verbal reinforce-ment | face-to-face sessions and telephone contact | Occupational Therapist (OT) | face-to-face, telephone | pre discharge at pt's bedside or quiet room, and on telephone | up to 3 x pre‐discharge, telephone contact up to 3 post-discharge (d/c) | choose topics from a list of 34 topics and the level of information detail | not reported | designed to be provided by any health care provider (HCP) with stroke knowledge/ experience |  |
| **Evans-Hudnall** 2014 | improving secondary stroke self-care | Cognitive Behavior Therapy (CBT) | nil outlined | face-to-face session and 2 biweekly telephone f/u | Research Assistant (RA) | face-to-face, telephone | in acute care and on telephone | 3 x 30 - 45 min 1 x pre-discharge 2 x biweekly over 4/52 after d/c | culturally tailored based on religiosity & collectivism. Free of charge | not reported | experienced health educator received additional training |  |
| **Gillham** 2010 | enhanced secondary prevention education | Motivational Interviewing (MI) | nil outlined | face-to-face session, telephone support & f/u | not reported | face-to-face, telephone | stroke clinic and on telephone | following randomisation & at 2/52 & 6/52 | not reported | not reported | not reported |  |
| **Green** 2007 | educational counselling intervention | Transtheoretical Stages of Change model with MI | a lifestyle class manual with print information | face-to-face motivational counselling & lifestyle class | nurse | individual and group | clinic | initial clinic visit (15 - 20 mins), class with 1 - 2/12 | individual risk factor assessment, strategies, readiness to change | not reported | study nurse with extensive experience |  |
| **Hjelle** 2019 | dialogue-based psychosocial wellbeing intervention | Salutogenesis; sense of coherence (SOC); narrative theory; self-determin-ation | themes & content worksheet | 8 individual sessions | research nurse / OT | face-to-face | participant's homes | 8 sessions 4 - 8 weeks post stroke, final session within 6/12 | related to patients' experiences | not reported | IP received 3 day training program | delivered according to protocol |
| **Hoffman** 2015 | enhancing early emotional adjustment following stroke | MI; self-efficacy | elements of each intervention outlined (2) education package & written info | 8 x 1hour sessions | (1) clinical psychologist (2) OT | face-to-face | 1st 2 sessions in hospital, then in pt's home | 1st 2 pre d/c, remaining within 2/12 | individually tailored | not reported | delivered by 1 experienced clinical psychologist and 1 OT |  |
| **Jones** 2016 | Bridges stroke Self Management Program (SMP) | social cognition theory & self-efficacy | based on 7 principles and a workbook | one-to-one individualised sessions | community stroke rehabilitation teams | face-to-face | community-based | integrated into scheduled rehabilitation sessions | individualised | not reported | feasibility, fidelity, acceptability included | found to be feasible & delivered as planned |
| **Kendall** 2007 | Stroke specific Chronic Disease SMP | Stanford model self-efficacy | 5 self-management principles informed small group education | 7 2hour group sessions | 2 trained health professionals | group face-to-face | community setting | 3 - 6 months post d/c over 6 weeks, 2 hours each week | stroke specific information session included | not reported | not reported |  |
| **Kootker** 2017 | augmented CBT for post-stroke depression v Computerized Cognitive Training (CCT) | CBT | individual sessions + OT/movement sessionsCCT Cogniplus program | 1 hour individual sessions | (1) psychologist, OT, movement therapist(2) self-administered with assistance of trainers | (1) face-to-face(2) desktop | rehabilitation unit | 4/12 period 13 - 16 sessions; each session divided with a break | (1) OT session atuned to psych session (2) individual level & pace | assessment timing adjusted due to lower inclusion rate |  |  |
| **McKenna** 2015 | Community based Bridges SMP Northern Ireland | self-efficacy principles | individual sessions workbook & vignettes | structured one-to-one sessions | community stroke team | face-to-face | community setting | one 1 hour session weekly x 6 weeks | content determined by pt personal goals, facilitated self-management practice | not reported | comprehensive training and practice for each professional | adherence defined and measured |
| **O Carroll** 2013 | increasing adherence to preventive medication | self-regulation theory; implementations intention approach | advice on medication taking, routines, behaviours, practice | 2 sessions to establish medication taking routine | research fellow | face-to-face | patient's home or hospital based research facility | session 1: about 3/12 post d/c session 2: 2 wks later; 2 sessions 2/52 apart; monthly MEMS pill bottle refill and electronic reading x 3/12 | individually tailored to establish routine | reviewed and modified collaboratively |  |  |
| **Sit** 2007 | community based prevention programme | self-care/self-managemnt | games, personal log sheets, pedometers | group education classes with experience sharing, individual goal setting, | community nurses | group face-to-face | community setting | groups of 10 - 12, 8 x 2 hour class, once a week x 8/52 | focused on individual goal setting and action planning | not reported | followed a structured validated protocol |  |
| **Sit** 2016 | health empowered stroke self-managemnt programme | theory of health empowerment;  self-efficacy; self-managemnt | group sessions, workbook, f/u & feedback | Commenced in wk 3 of rehab schedule. Part 1: in parallel with ambulatory rehabilitation Part 2: home-based implementation with telephone f/u | nurse facilitator | groups of 4-6 face-to-face, telephone f/u | ambulatory rehabilitation centre | small group sessions weekly from wk 3-8 of rehab; biweekly telephone f/u weeks 9-13 | establish partnership, personal goal setting, individual feedback | not reported | followed a structured validated protocol; Protocol driven telephone f/u |  |
| **Slark** 2013 | individual risk awareness intervention | Effectiveness of an intervention to increase awareness of risk and stroke knowledge | individual risk factor information and risk score | risk awareness intervention session guided by a proforma | not reported | face-to-face | in acute care pre discharge | individual 30 minute session | focused on individual risk factorawareness information | not reported | intervention proforma provided |  |
| **Tielemans** 2015 | Restore4stroke self-managemnt intervention "Plan ahead" | proactive coping based self-management vs education | guides, presentations, workbook | small groups & booster sessions teaching proactive planning | HCPs | group face-to-face, at least 6/52 after stroke | outpatient facilities | I(1):10 weeks, 7 x 2hour group sessionsI(2): 10 weeks, 4 x 1hour group sessions |  | not reported | at least 8 recruited at each site, | different HCPs assigned and intervention specific trained |
| **Twofighi** 2020 | lifestyle management programme | TTT readiness to change; health beliefs model; social cognitive or learning theory;  self-efficacy | Manual with handouts and action plans; calorie-counting, physical activity, weight logs; pedometers | didactic presentations, peer exchange, personal exploration, healthy lifestyle activities | OTs | group | rehabilitation centre | 6 weekly 2-hour sessions | conducted in english or spanish | adapted from 'Lifestyle Redesign' approach, based on national guidelines | a small pilot trial with 9 participants to gauge feasibility. 3 additional Ots trained to deliver intervention | feasible and well received. Formative evaluation contributed to informed development and recommendations |
| **Wang** 2020 | effects of Mindfulness Based Intervention on quality of life and poststroke depression in patients with sICH in China | Mindfullness Based Cognitive Therapy (MBCT) | not reported | therapists provided support to participants in learning to respond adaptively to thoughts, feelings, and experiences | 3 professional therapists | group | hospital | 8 2-hour group sessions over consecutive weeks | not reported | not reported | control group received 8/52 stress management education sessions | supported the effectiveness of mindfulness meditation, but positive effects could be attributed to traditional CBT |
| **Watkins** 2007 & 2011 | Early MI to improve mood | MI talk therapy | individual sessions with therapist | therapist set agenda to talk about adjustments,elicited goals, perceived blocks, enabled own solutions | therapists (nursing & non-clinical psychology) | face-to-face | in-hospital in a private area | up to 4 sessions, 1 per week, lasting 30-60 mins | individual goal setting, pt's dilemmas, ambivalence, supported self-efficacy | not reported | 4/7 training, 10 practice sessions, supervised by psychologist,. Sessions recorded for learning | quality assessment of MI application |
| **Zhang** 2015 | clinical effects of mindfulness-based bahaviour in post-stroke depression | MBCT | group practice | instruction and practice, relaxation training, shared experience and discussion | trained psychological therapists | group of 5 - 8 people | psychotherapy centre | 8/52, 90 - 120mins x 3 times a week | not reported | not reported | followed a specific 4 stage treatment plan | depression scores decreased in both groups, MBCT group performed better |
