# Supplementary material for: Reducing risk behaviours after stroke: An overview of reviews interrogating primary study data using the Theoretical Domains Framework
Source: PLoS One. 2024 Apr 26;19(4):e0302364. doi: 10.1371/journal.pone.0302364 (PMC11051587; doi:10.1371/journal.pone.0302364)
Supplement: S1 File — (DOCX) [file pone.0302364.s005.docx]

**S1 File. Search Strategy**

**MEDLINE (OVID)**

**Stroke**

1. cerebrovascular disorders/ or exp basal ganglia cerebrovascular disease/ or exp brain ischemia/ or exp carotid artery diseases/ or exp intracranial arterial diseases/ or exp intracranial arteriovenous malformations/ or exp "intracranial embolism and thrombosis"/ or exp intracranial hemorrhages/ or stroke/ or exp brain infarction/ or vasospasm, intracranial/ or vertebral artery dissection/

2. (stroke or poststroke or post-stroke or cerebrovasc$ or brain vasc$ or cerebral vasc$ or cva$ or apoplex$ or SAH or TIA or transient isch?emic attack or vertebral artery dissection).tw.

3. ((brain$ or cerebr$ or cerebell$ or intracran$ or intracerebral) adj5 (isch?emi$ or infarct$ or thrombo$ or emboli$ or occlus$ or disorder$)).tw.

4. ((brain$ or cerebr$ or cerebell$ or intracerebral or intracranial or subarachnoid) adj5 (haemorrhage$ or hemorrhage$ or haematoma$ or hematoma$ or bleed$)).tw.

5. 1 or 2 or 3 or 4

**Risk reduction**

6. exp Health Education/ or exp Health Promotion/ or exp Health Behavior/ or exp Secondary Prevention/ or exp Counseling/

7. (health education or health promotion or health behavior or secondary prevention or counseling or counsel$).mp.

8. (health adj5 (educat$ or program$ or promotion$ or behavio?r)).tw.

9. (patient adj5 (educat$ or program$)).tw.

10. 6 or 7 or 8 or 9

11. ((secondary or multifactor$) adj3 (prevention or intervention)).tw.

12. (risk adj3 factor$ adj5 (reduc$ or manag$ or intervent$)).tw.

13. (lifestyle adj3 (intervent$ or advice)).tw.

14. (life?style adj3 (intervention$ or advice or alter$ or educat$ or chang$)).tw.

15. (behavio?r$ adj3 chang$).tw.

16. (health?care adj3 advice).tw.

17. non?pharmacologic$.tw.

18. 11 or 12 or 13 or 14 or 15 or 16 or 17

19. ethanol.mp. or exp Ethanol/

20. (alcohol$ or ethanol$ or wine or beer or spirit$ or ((problem or hazardous or harmful) adj3 drink$)).tw.

21. 19 or 20

22. exp Tobacco/ or "Tobacco Use Cessation"/ or exp smoking/ or exp smoking cessation/

23. tobacco.mp.

24. (tobacco or smok$).tw.

25. 22 or 23 or 24

26. diet$.tw.

27. (healthy adj3 eating).tw.

28. (diet adj3 chang$).tw.

29. 26 or 27 or 28

30. exercise.mp. or exp Exercise/

31. (physical adj3 activ$).tw.

32. 30 or 31

33. 10 or 18 or 21 or 25 or 29 or 32

34. 5 and 33

35. limit 34 to humans

**Systematic reviews**

36. meta-analysis/ or literature review/

37. systematic review.pt.

38. meta?analy$.tw.

39. ((systematic or quantitative or methodolog$) adj (overview$ or review$)).tw.

40. integrative research review$.tw.

41. 36 or 37 or 38 or 39 or 40

**42. 35 and 41**

**EMBASE (Platform)**

#1 'cerebrovascular disease'/de OR 'basal ganglion hemorrhage'/exp OR 'brain ischemia'/exp OR 'carotid artery disease'/exp OR 'cerebral artery disease'/exp OR 'brain arteriovenous malformation'/exp OR 'brain embolism'/exp OR 'occlusive cerebrovascular disease'/exp OR 'brain hemorrhage'/exp OR 'cerebrovascular accident'/exp OR 'brain infarction'/exp OR 'brain vasospasm'/exp OR 'artery dissection'/exp

#2 stroke:ti,ab,kw OR poststroke:ti,ab,kw OR 'post stroke':ti,ab,kw OR cerebrovsc*:ti,ab,kw OR 'brain vasc*':ti,ab,kw OR 'cerebral vasc*':ti,ab,kw OR cva*:ti,ab,kw OR apoplex*:ti,ab,kw OR sah:ti,ab,kw OR tia:ti,ab,kw OR 'transient ischaemic attack':ti,ab,kw OR 'transient ischemic attack':ti,ab,kw OR 'vertebral artery dissection':ti,ab,kw

#3 ((brain* OR cerebr* OR cerebell* OR intracran* OR intracerebral) NEAR/5 (ischemi* OR ischaemi* OR infarct* OR thrombo* OR emboli* OR occlus* OR disorder*)):ti,ab,kw

#4 ((brain* OR cerebr* OR cerebell* OR intracerebral OR intracranial OR subarachnoid) NEAR/5 (haemorrhage* OR hemorrhage* OR haematoma* OR hematoma* OR bleed*)):ti,ab,kw

**#5 #1 OR #2 OR #3 OR #4**

#6 'health education'/exp OR 'health promotion'/exp OR 'health behavior'/exp OR 'secondary prevention'/exp OR 'counseling'/exp

#7 'health education':kw OR 'health promotion':kw OR 'health behavior':kw OR 'health behaviour':kw OR 'secondary prevention':kw OR counseling:kw OR counsel*:kw

#8 ((health NEAR/5 (educat* OR program* OR promotion* OR behavior OR behaviour))):ti,ab,kw

#9 ((patient NEAR/5 (educat* or program*))):ti,ab,kw

**#10** #6 OR #7 OR #8 OR #9

#11 ((secondary OR multifactor*) NEAR/3 (prevention OR intervention)):ti,ab,kw

#12 (risk NEAR/3 factor* NEAR/5 (reduc* OR manag* OR intervent*)):ti,ab,kw

#13 (lifestyle NEAR/3 (intervent* OR advice)):ti,ab,kw

#14 ('life style' NEAR/3 (intervention* OR advice OR alter* OR educat* OR chang*)):ti,ab,kw

#15 (behavior* OR behaviour*) NEAR/3 chang*):ti,ab,kw

#16 (('health care' OR healthcare) NEAR/3 advice):ti,ab,kw

#17 'non pharmacologic*':ti,ab,kw OR nonpharmacologic*:ti,ab,kw

**#18** #11 OR #12 OR #13 OR #14 OR #15 OR #16 OR #17

#19 'alcohol'/exp OR 'alcohol'

#20 alcohol*:ti,ab,kw OR ethanol*:ti,ab,kw OR wine:ti,ab,kw OR beer:ti,ab,kw OR spirit*:ti,ab,kw OR (((problem OR hazardous OR harmful) NEAR/3 drink*):ti,ab,kw)

**#21** #19 OR #20

#22 'tobacco'/exp OR 'smoking cessation'/exp OR 'smoking'/exp OR 'tobacco use'/exp

#23 tobacco:kw

#24 tobacco:ti,ab,kw OR smok*:ti,ab,kw

**#25** #22 OR #23 OR #24

#26 diet*:ti,ab,kw

#27 (healthy NEAR/3 eating):ti,ab,kw

#28 (diet NEAR/3 chang*):ti,ab,kw

**#29** #26 OR #27 OR #28

#30 'exercise'/exp OR exercise:kw

#31 (physical NEAR/3 activ*):ti,ab,kw

**#32** #30 OR #31

**#33** #10 OR #18 OR #21 OR #25 OR #29 OR #32

**#34** #5 AND #33

#35 #5 AND #33 AND [humans]/lim

#36 'meta analysis'/exp OR 'literature review'/exp

#37 'systematic review (topic)'

#38 'meta analysis'/exp OR 'meta analysis'

#39 (systematic:ti,ab,kw OR quantitative:ti,ab,kw OR methodolog*:ti,ab,kw) AND (overview*:ti,ab,kw OR review*:ti,ab,kw)

#40 'integrative research review*':ti,ab,kw

**#41** #36 OR #37 OR #38 OR #39 OR #40

**#42 #35 AND #41**

**Epistemonikos**

**stroke**

1. (title:(stroke OR poststroke OR post-stroke OR cerebrovasc* OR "brain vasc*" OR "CVA" OR apoplex* OR "SAH" OR "TIA" OR "transient ischemic attack" OR "transient ischaemic attack" OR "vertebral artery dissection") OR abstract:(stroke OR poststroke OR post-stroke OR cerebrovasc* OR "brain vasc*" OR "CVA" OR apoplex* OR "SAH" OR "TIA" OR "transient ischemic attack" OR "transient ischaemic attack" OR "vertebral artery dissection"))

2. (title:((brain* OR cerebr* OR cerebell* OR intracerebral OR intracranial OR subarachnoid) AND (haemorrhage* OR hemorrhage* OR haematoma* OR hematoma* OR bleed*)) OR abstract:((brain* OR cerebr* OR cerebell* OR intracerebral OR intracranial OR subarachnoid) AND (haemorrhage* OR hemorrhage* OR haematoma* OR hematoma* OR bleed*)))

3. (title:((brain* OR cerebr* OR cerebell* OR intracerebral OR intracranial OR subarachnoid) AND (ischemi* OR ischaemi* OR infarct* OR thrombo* OR emboli* OR occlus* OR disorder)) OR abstract:((brain* OR cerebr* OR cerebell* OR intracerebral OR intracranial OR subarachnoid) AND (ischemi* OR ischaemi* OR infarct* OR thrombo* OR emboli* OR occlus* OR disorder)))

4. 1 OR 2 OR 3 (title:((brain* OR cerebr* OR cerebell* OR intracerebral OR intracranial OR subarachnoid) AND (ischemi* OR ischaemi* OR infarct* OR thrombo* OR emboli* OR occlus* OR disorder)) OR abstract:((brain* OR cerebr* OR cerebell* OR intracerebral OR intracranial OR subarachnoid) AND (ischemi* OR ischaemi* OR infarct* OR thrombo* OR emboli* OR occlus* OR disorder))) OR (title:((brain* OR cerebr* OR cerebell* OR intracerebral OR intracranial OR subarachnoid) AND (ischemi* OR ischaemi* OR infarct* OR thrombo* OR emboli* OR occlus* OR disorder)) OR abstract:((brain* OR cerebr* OR cerebell* OR intracerebral OR intracranial OR subarachnoid) AND (ischemi* OR ischaemi* OR infarct* OR thrombo* OR emboli* OR occlus* OR disorder))) OR (title:(stroke) OR abstract:(stroke)) OR (title:(poststroke) OR abstract:(poststroke)) OR (title:(post-stroke) OR abstract:(post-stroke)) OR (title:(cerebrovasc*) OR abstract:(cerebrovasc*)) OR (title:("brain vasc*") OR abstract:("brain vasc*")) OR (title:(CVA*) OR abstract:(CVA*)) OR (title:(apoplex*) OR abstract:(apoplex*)) OR (title:(SAH) OR abstract:(SAH)) OR (title:(TIA) OR abstract:(TIA)) OR (title:("transient ischaemic attack" OR "transient ischemic attack") OR abstract:("transient ischaemic attack" OR "transient ischemic attack")) OR (title:("vertebral artery dissection") OR abstract:("vertebral artery dissection"))

(title:(stroke OR poststroke OR post-stroke OR cerebrovasc* OR "brain vasc*" OR "CVA" OR apoplex* OR "SAH" OR "TIA" OR "transient ischemic attack" OR "transient ischaemic attack" OR "vertebral artery dissection") OR abstract:(stroke OR poststroke OR post-stroke OR cerebrovasc* OR "brain vasc*" OR "CVA" OR apoplex* OR "SAH" OR "TIA" OR "transient ischemic attack" OR "transient ischaemic attack" OR "vertebral artery dissection")) OR (title:(((brain* OR cerebr* OR cerebell* OR intracerebral OR intracranial OR subarachnoid) AND (haemorrhage* OR hemorrhage* OR haematoma* OR hematoma* OR bleed*))) OR abstract:(((brain* OR cerebr* OR cerebell* OR intracerebral OR intracranial OR subarachnoid) AND (haemorrhage* OR hemorrhage* OR haematoma* OR hematoma* OR bleed*)))) OR (title:(((brain* OR cerebr* OR cerebell* OR intracerebral OR intracranial OR subarachnoid) AND (ischemi* OR ischaemi* OR infarct* OR thrombo* OR emboli* OR occlus* OR disorder))) OR abstract:(((brain* OR cerebr* OR cerebell* OR intracerebral OR intracranial OR subarachnoid) AND (ischemi* OR ischaemi* OR infarct* OR thrombo* OR emboli* OR occlus* OR disorder))))

**Risk**

5. (title:("health education" OR "health promotion" OR "health behavior" OR "health behaviour" OR "secondary prevention" OR counseling OR counsel*) OR abstract:("health education" OR "health promotion" OR "health behavior" OR "health behaviour" OR "secondary prevention" OR counseling OR counsel*))

6. (title:(health AND (educat* OR program* OR promotion* OR behavior OR behaviour)) OR abstract:(health AND (educat* OR program* OR promotion* OR behavior OR behaviour)))

7. (title:(patient AND (educat* OR program*)) OR abstract:(patient AND (educat* OR program*)))

8. 5 OR 6 OR 7 (title:(patient AND (educat* OR program*)) OR abstract:(patient AND (educat* OR program*))) OR (title:(health AND (educat* OR program* OR promotion* OR behavior OR behaviour)) OR abstract:(health AND (educat* OR program* OR promotion* OR behavior OR behaviour))) OR (title:("health education" OR "health promotion" OR "health behavior" OR "health behaviour" OR "secondary prevention" OR counseling OR counsel*) OR abstract:("health education" OR "health promotion" OR "health behavior" OR "health behaviour" OR "secondary prevention" OR counseling OR counsel*))

9. (title:(((secondary OR multifactor*) AND (prevention OR intervention))) OR abstract:(((secondary OR multifactor*) AND (prevention OR intervention))))

10. (title:(risk factor* AND (reduc* OR manag* OR intervent*)) OR abstract:(risk factor* AND (reduc* OR manag* OR intervent*)))

11. (title:((lifestyle OR life-style) AND (intervent* OR advice OR alter* OR educat* OR chang*)) OR abstract:((lifestyle OR life-style) AND (intervent* OR advice OR alter* OR educat* OR chang*)))

12. (title:((behavior* OR behaviour*) AND chang*) OR abstract:((behavior* OR behaviour*) AND chang*))

13. (title:((health care OR healthcare) AND advice) OR abstract:((health care OR healthcare) AND advice))

14. (title:(non-pharmacologic* OR nonpharmacologic*) OR abstract:(non-pharmacologic* OR nonpharmacologic*))

15. 9 OR 10 OR 11 OR 12 OR 13 OR 14 (title:(non-pharmacologic* OR nonpharmacologic*) OR abstract:(non-pharmacologic* OR nonpharmacologic*)) OR (title:((health care OR healthcare) AND advice) OR abstract:((health care OR healthcare) AND advice)) OR (title:((behavior* OR behaviour*) AND chang*) OR abstract:((behavior* OR behaviour*) AND chang*)) OR (title:((lifestyle OR life-style) AND (intervent* OR advice OR alter* OR educat* OR chang*)) OR abstract:((lifestyle OR life-style) AND (intervent* OR advice OR alter* OR educat* OR chang*))) OR (title:(risk factor* AND (reduc* OR manag* OR intervent*)) OR abstract:(risk factor* AND (reduc* OR manag* OR intervent*))) OR (title:(((secondary OR multifactor*) AND (prevention OR intervention))) OR abstract:(((secondary OR multifactor*) AND (prevention OR intervention))))

16. (title:(ethanol OR alcohol) OR abstract:(ethanol OR alcohol))

17. (title:(((alcohol* OR ethanol* OR wine OR beer OR spirit* OR (problem OR hazardous OR harmful)) AND drink*)) OR abstract:(((alcohol* OR ethanol* OR wine OR beer OR spirit* OR (problem OR hazardous OR harmful)) AND drink*)))

18. (title:(tobacco OR smok* OR "tobacco use cessation" OR "smoking cessation") OR abstract:(tobacco OR smok* OR "tobacco use cessation" OR "smoking cessation"))

19. (title:(diet* OR "healthy eating" OR (healthy AND eating) OR (diet AND chang*)) OR abstract:(diet* OR "healthy eating" OR (healthy AND eating) OR (diet AND chang*)))

20. (title:(exercise OR "physical activ*" OR (physical AND activ*)) OR abstract:(exercise OR "physical activ*" OR (physical AND activ*)))

21. 15 OR 16 OR 17 OR 18 OR 19 OR 20 (title:(exercise OR "physical activ*" OR (physical AND activ*)) OR abstract:(exercise OR "physical activ*" OR (physical AND activ*))) OR (title:(diet* OR "healthy eating" OR (healthy AND eating) OR (diet AND chang*)) OR abstract:(diet* OR "healthy eating" OR (healthy AND eating) OR (diet AND chang*))) OR (title:(tobacco OR smok* OR "tobacco use cessation" OR "smoking cessation") OR abstract:(tobacco OR smok* OR "tobacco use cessation" OR "smoking cessation")) OR (title:(((alcohol* OR ethanol* OR wine OR beer OR spirit* OR (problem OR hazardous OR harmful)) AND drink*)) OR abstract:(((alcohol* OR ethanol* OR wine OR beer OR spirit* OR (problem OR hazardous OR harmful)) AND drink*))) OR (title:(ethanol OR alcohol) OR abstract:(ethanol OR alcohol))

33. **Risk reduction AND Stroke**

(title: (("health education" OR "health promotion" OR "health behavior" OR "health behaviour" OR "secondary prevention" OR counseling OR counsel*)) OR abstract:(("health education" OR "health promotion" OR "health behavior" OR "health behaviour" OR "secondary prevention" OR counseling OR counsel*))) OR (title: ((health AND (educat* OR program* OR promotion* OR behavior OR behaviour))) OR abstract:((health AND (educat* OR program* OR promotion* OR behavior OR behaviour)))) OR (title: ((patient AND (educat* OR program*))) OR abstract:((patient AND (educat* OR program*)))) OR (title: (((secondary OR multifactor*) AND (prevention OR intervention))) OR abstract:(((secondary OR multifactor*) AND (prevention OR intervention)))) OR (title: ((risk factor* AND (reduc* OR manag* OR intervent*))) OR abstract:((risk factor* AND (reduc* OR manag* OR intervent*)))) OR (title: (((lifestyle OR life-style) AND (intervent* OR advice OR alter* OR educat* OR chang*))) OR abstract:(((lifestyle OR life-style) AND (intervent* OR advice OR alter* OR educat* OR chang*)))) OR (title: (((behavior* OR behaviour*) AND chang*)) OR abstract:(((behavior* OR behaviour*) AND chang*))) OR (title: ((health care OR healthcare) AND advice) OR abstract:((health care OR healthcare) AND advice)) OR (title: (non-pharmacologic* OR nonpharmacologic*) OR abstract:(non-pharmacologic* OR nonpharmacologic*)) OR (title: (ethanol OR alcohol) OR abstract:(ethanol OR alcohol)) OR (title: ((alcohol* OR ethanol* OR wine OR beer OR spirit* OR (problem OR hazardous OR harmful)) AND drink*) OR abstract:((alcohol* OR ethanol* OR wine OR beer OR spirit* OR (problem OR hazardous OR harmful)) AND drink*)) OR (title: (tobacco OR smok* OR "tobacco use cessation" OR "smoking cessation") OR abstract:(tobacco OR smok* OR "tobacco use cessation" OR "smoking cessation")) OR (title: (diet* OR "healthy eating" OR (healthy AND eating) OR (diet AND chang*)) OR abstract:(diet* OR "healthy eating" OR (healthy AND eating) OR (diet AND chang*))) OR (title: (exercise OR "physical activ*" OR (physical AND activ*)) OR abstract:(exercise OR "physical activ*" OR (physical AND activ*)))

AND

(title:(stroke OR poststroke OR post-stroke OR cerebrovasc* OR "brain vasc*" OR "CVA" OR apoplex* OR "SAH" OR "TIA" OR "transient ischemic attack" OR "transient ischaemic attack" OR "vertebral artery dissection") OR abstract:(stroke OR poststroke OR post-stroke OR cerebrovasc* OR "brain vasc*" OR "CVA" OR apoplex* OR "SAH" OR "TIA" OR "transient ischemic attack" OR "transient ischaemic attack" OR "vertebral artery dissection")) OR (title:(((brain* OR cerebr* OR cerebell* OR intracerebral OR intracranial OR subarachnoid) AND (haemorrhage* OR hemorrhage* OR haematoma* OR hematoma* OR bleed*))) OR abstract:(((brain* OR cerebr* OR cerebell* OR intracerebral OR intracranial OR subarachnoid) AND (haemorrhage* OR hemorrhage* OR haematoma* OR hematoma* OR bleed*)))) OR (title:(((brain* OR cerebr* OR cerebell* OR intracerebral OR intracranial OR subarachnoid) AND (ischemi* OR ischaemi* OR infarct* OR thrombo* OR emboli* OR occlus* OR disorder))) OR abstract:(((brain* OR cerebr* OR cerebell* OR intracerebral OR intracranial OR subarachnoid) AND (ischemi* OR ischaemi* OR infarct* OR thrombo* OR emboli* OR occlus* OR disorder))))

**Stroke**

stroke OR poststroke OR post-stroke OR cerebrovasc* OR "brain vasc*" OR "CVA" OR apoplex* OR "SAH" OR "TIA" OR "transient ischemic attack" OR "transient ischaemic attack" OR "vertebral artery dissection" OR ((brain* OR cerebr* OR cerebell* OR intracerebral OR intracranial OR subarachnoid) AND (haemorrhage* OR hemorrhage* OR haematoma* OR hematoma* OR bleed*)) OR ((brain* OR cerebr* OR cerebell* OR intracerebral OR intracranial OR subarachnoid) AND (ischemi* OR ischaemi* OR infarct* OR thrombo* OR emboli* OR occlus* OR disorder))

**Risk reduction**

(("health education" OR "health promotion" OR "health behavior" OR "health behaviour" OR "secondary prevention" OR counseling OR counsel*)) OR ((health AND (educat* OR program* OR promotion* OR behavior OR behaviour))) OR ((patient AND (educat* OR program*))) OR (((secondary OR multifactor*) AND (prevention OR intervention))) OR ((risk factor* AND (reduc* OR manag* OR intervent*))) OR (((lifestyle OR life-style) AND (intervent* OR advice OR alter* OR educat* OR chang*))) OR (((behavior* OR behaviour*) AND chang*)) OR ((health care OR healthcare) AND advice) OR (non-pharmacologic* OR nonpharmacologic*) OR (ethanol OR alcohol) OR ((alcohol* OR ethanol* OR wine OR beer OR spirit* OR (problem OR hazardous OR harmful)) AND drink*) OR (tobacco OR smok* OR "tobacco use cessation" OR "smoking cessation") OR (diet* OR "healthy eating" OR (healthy AND eating) OR (diet AND chang*)) OR (exercise OR "physical activ*" OR (physical AND activ*))

**Combined using AND, limited to Title/Abstract**

(title:((title:(stroke OR poststroke OR post-stroke OR cerebrovasc* OR "brain vasc*" OR "CVA" OR apoplex* OR "SAH" OR "TIA" OR "transient ischemic attack" OR "transient ischaemic attack" OR "vertebral artery dissection" OR ((brain* OR cerebr* OR cerebell* OR intracerebral OR intracranial OR subarachnoid) AND (haemorrhage* OR hemorrhage* OR haematoma* OR hematoma* OR bleed*)) OR ((brain* OR cerebr* OR cerebell* OR intracerebral OR intracranial OR subarachnoid) AND (ischemi* OR ischaemi* OR infarct* OR thrombo* OR emboli* OR occlus* OR disorder))) OR abstract:(stroke OR poststroke OR post-stroke OR cerebrovasc* OR "brain vasc*" OR "CVA" OR apoplex* OR "SAH" OR "TIA" OR "transient ischemic attack" OR "transient ischaemic attack" OR "vertebral artery dissection" OR ((brain* OR cerebr* OR cerebell* OR intracerebral OR intracranial OR subarachnoid) AND (haemorrhage* OR hemorrhage* OR haematoma* OR hematoma* OR bleed*)) OR ((brain* OR cerebr* OR cerebell* OR intracerebral OR intracranial OR subarachnoid) AND (ischemi* OR ischaemi* OR infarct* OR thrombo* OR emboli* OR occlus* OR disorder)))) AND (title:((("health education" OR "health promotion" OR "health behavior" OR "health behaviour" OR "secondary prevention" OR counseling OR counsel*)) OR ((health AND (educat* OR program* OR promotion* OR behavior OR behaviour))) OR ((patient AND (educat* OR program*))) OR (((secondary OR multifactor*) AND (prevention OR intervention))) OR ((risk factor* AND (reduc* OR manag* OR intervent*))) OR (((lifestyle OR life-style) AND (intervent* OR advice OR alter* OR educat* OR chang*))) OR (((behavior* OR behaviour*) AND chang*)) OR ((health care OR healthcare) AND advice) OR (non-pharmacologic* OR nonpharmacologic*) OR (ethanol OR alcohol) OR ((alcohol* OR ethanol* OR wine OR beer OR spirit* OR (problem OR hazardous OR harmful)) AND drink*) OR (tobacco OR smok* OR "tobacco use cessation" OR "smoking cessation") OR (diet* OR "healthy eating" OR (healthy AND eating) OR (diet AND chang*)) OR (exercise OR "physical activ*" OR (physical AND activ*))) OR abstract:((("health education" OR "health promotion" OR "health behavior" OR "health behaviour" OR "secondary prevention" OR counseling OR counsel*)) OR ((health AND (educat* OR program* OR promotion* OR behavior OR behaviour))) OR ((patient AND (educat* OR program*))) OR (((secondary OR multifactor*) AND (prevention OR intervention))) OR ((risk factor* AND (reduc* OR manag* OR intervent*))) OR (((lifestyle OR life-style) AND (intervent* OR advice OR alter* OR educat* OR chang*))) OR (((behavior* OR behaviour*) AND chang*)) OR ((health care OR healthcare) AND advice) OR (non-pharmacologic* OR nonpharmacologic*) OR (ethanol OR alcohol) OR ((alcohol* OR ethanol* OR wine OR beer OR spirit* OR (problem OR hazardous OR harmful)) AND drink*) OR (tobacco OR smok* OR "tobacco use cessation" OR "smoking cessation") OR (diet* OR "healthy eating" OR (healthy AND eating) OR (diet AND chang*)) OR (exercise OR "physical activ*" OR (physical AND activ*))))) OR abstract:((title:(stroke OR poststroke OR post-stroke OR cerebrovasc* OR "brain vasc*" OR "CVA" OR apoplex* OR "SAH" OR "TIA" OR "transient ischemic attack" OR "transient ischaemic attack" OR "vertebral artery dissection" OR ((brain* OR cerebr* OR cerebell* OR intracerebral OR intracranial OR subarachnoid) AND (haemorrhage* OR hemorrhage* OR haematoma* OR hematoma* OR bleed*)) OR ((brain* OR cerebr* OR cerebell* OR intracerebral OR intracranial OR subarachnoid) AND (ischemi* OR ischaemi* OR infarct* OR thrombo* OR emboli* OR occlus* OR disorder))) OR abstract:(stroke OR poststroke OR post-stroke OR cerebrovasc* OR "brain vasc*" OR "CVA" OR apoplex* OR "SAH" OR "TIA" OR "transient ischemic attack" OR "transient ischaemic attack" OR "vertebral artery dissection" OR ((brain* OR cerebr* OR cerebell* OR intracerebral OR intracranial OR subarachnoid) AND (haemorrhage* OR hemorrhage* OR haematoma* OR hematoma* OR bleed*)) OR ((brain* OR cerebr* OR cerebell* OR intracerebral OR intracranial OR subarachnoid) AND (ischemi* OR ischaemi* OR infarct* OR thrombo* OR emboli* OR occlus* OR disorder)))) AND (title:((("health education" OR "health promotion" OR "health behavior" OR "health behaviour" OR "secondary prevention" OR counseling OR counsel*)) OR ((health AND (educat* OR program* OR promotion* OR behavior OR behaviour))) OR ((patient AND (educat* OR program*))) OR (((secondary OR multifactor*) AND (prevention OR intervention))) OR ((risk factor* AND (reduc* OR manag* OR intervent*))) OR (((lifestyle OR life-style) AND (intervent* OR advice OR alter* OR educat* OR chang*))) OR (((behavior* OR behaviour*) AND chang*)) OR ((health care OR healthcare) AND advice) OR (non-pharmacologic* OR nonpharmacologic*) OR (ethanol OR alcohol) OR ((alcohol* OR ethanol* OR wine OR beer OR spirit* OR (problem OR hazardous OR harmful)) AND drink*) OR (tobacco OR smok* OR "tobacco use cessation" OR "smoking cessation") OR (diet* OR "healthy eating" OR (healthy AND eating) OR (diet AND chang*)) OR (exercise OR "physical activ*" OR (physical AND activ*))) OR abstract:((("health education" OR "health promotion" OR "health behavior" OR "health behaviour" OR "secondary prevention" OR counseling OR counsel*)) OR ((health AND (educat* OR program* OR promotion* OR behavior OR behaviour))) OR ((patient AND (educat* OR program*))) OR (((secondary OR multifactor*) AND (prevention OR intervention))) OR ((risk factor* AND (reduc* OR manag* OR intervent*))) OR (((lifestyle OR life-style) AND (intervent* OR advice OR alter* OR educat* OR chang*))) OR (((behavior* OR behaviour*) AND chang*)) OR ((health care OR healthcare) AND advice) OR (non-pharmacologic* OR nonpharmacologic*) OR (ethanol OR alcohol) OR ((alcohol* OR ethanol* OR wine OR beer OR spirit* OR (problem OR hazardous OR harmful)) AND drink*) OR (tobacco OR smok* OR "tobacco use cessation" OR "smoking cessation") OR (diet* OR "healthy eating" OR (healthy AND eating) OR (diet AND chang*)) OR (exercise OR "physical activ*" OR (physical AND activ*))))))

**Limited to systematic reviews**

**Cochrane Library**

1. (MeSH descriptor: [Cerebrovascular Disorders] this term only OR MeSH descriptor: [Basal Ganglia Cerebrovascular Disease] explode all trees OR MeSH descriptor: [Brain Ischemia] explode all trees OR MeSH descriptor: [Carotid Artery Diseases] explode all trees OR MeSH descriptor: [Intracranial Arterial Diseases] explode all trees OR MeSH descriptor: [Intracranial Arteriovenous Malformations] explode all trees OR MeSH descriptor: [Intracranial Embolism and Thrombosis] explode all trees OR MeSH descriptor: [Intracranial Hemorrhages] explode all trees OR MeSH descriptor: [Stroke] this term only OR MeSH descriptor: [Brain Infarction] explode all trees OR MeSH descriptor: [Vasospasm, Intracranial] this term only OR MeSH descriptor: [Vertebral Artery Dissection] this term only)

(#1 OR #2 OR #3 OR #4 OR #5 OR #6 OR #7 OR #8 OR #9 OR #10 OR #11 OR #12 **= #13**)

2. ((stroke OR poststroke OR post-stroke OR cerebrovsc* OR brain vasc* OR cerebral vasc* OR CVA* OR apoplex* OR SAH OR TIA OR transient ischaemic attack OR transient ischemic attack OR vertebral artery dissection)):ti,ab,kw (#14)

3. ((brain* OR cerebr* OR cerebell* OR intracran* OR intracerebral) NEAR/5 (ischemi* OR ischaemi* OR infarct* OR thrombo* OR emboli* OR occlus*)):ti,ab,kw (#15)

4. ((brain* OR cerebr* OR cerebell* OR intracerebral OR intracranial OR subarachnoid) NEAR/5 (haemorrhage* OR hemorrhage* OR haematoma* OR hematoma* OR bleed*)):ti,ab,kw (#16)

5. #13 OR #14 OR #15 OR #16 = **#17**

6. (MeSH descriptor: [Health Education] explode all trees OR MeSH descriptor: [Health Promotion] explode all trees OR MeSH descriptor: [Health Behavior] explode all trees OR MeSH descriptor: [Secondary Prevention] explode all trees OR MeSH descriptor: [Counseling] explode all trees) (#18 OR #19 OR #20 OR #21 OR #22) = #23

7. ((health education OR health promotion OR health behavior OR health behaviour OR secondary prevention OR counseling OR counsel*)):kw (#24)

8. ((health NEAR/5 (educat* OR program* OR promotion* OR behavior OR behaviour))):ti,ab,kw (#25)

9. ((patient NEAR/5 (educat* or program*))):ti,ab,kw (#26)

10. #23 OR #24 OR #25 OR #26 = **#27**

11. (((secondary or multifactor*) NEAR/3 (prevention or intervention))):ti,ab,kw (#28)

12. (((risk NEAR/3 factor* NEAR/5 (reduc* OR manag* OR intervent*)))):ti,ab,kw (#29)

13. ((lifestyle NEAR/3 (intervent* or advice))):ti,ab,kw (#30)

14. (((life style NEAR/3 (intervention* OR advice OR alter* OR educat* OR chang*)))):ti,ab,kw (#31)

15. ((((behavior* OR behaviour*) NEAR/3 chang*))):ti,ab,kw (#32)

16. ((((health care OR healthcare) NEAR/3 advice))):ti,ab,kw (#33)

17. ((non-pharmacologic* OR nonpharmacologic*)):ti,ab,kw (#34)

18. #28 OR #29 OR #30 OR #31 OR #32 OR #33 OR #34 **= #35**

19. ((ethanol):kw) OR (MeSH descriptor: [Ethanol] explode all trees)

(#36 OR #37)

20. (((alcohol* OR ethanol* OR wine OR beer OR spirit* or ((problem or hazardous or harmful) NEAR/3 drink*)))):ti,ab,kw (#38)

21. #36 OR #37 OR #38 = **#39**

22. (MeSH descriptor: [Tobacco] explode all trees OR MeSH descriptor: [Tobacco Use Cessation] explode all trees OR MeSH descriptor: [Smoking] explode all trees OR MeSH descriptor: [Smoking Cessation] explode all trees) (#40 OR #41 OR #42 OR #43) =#44

23. (tobacco):kw

24. (((tobacco OR smok*))):ti,ab,kw

25. (#44 OR #45 OR #46) = **#47**

26. (diet*):ti,ab,kw #48

27. (((healthy NEAR/3 eating))):ti,ab,kw #49

28. (((diet NEAR/3 chang*))):ti,ab,kw #50

29. #48 OR #49 OR #50 = **#51**

30. ((exercise):kw) OR (MeSH descriptor: [Exercise] explode all trees) #52 OR #53

31. (((physical NEAR/3 activ*))):ti,ab,kw

32. #52 OR #53 OR #54 = #55

33. #27 OR #35 OR #39 OR #47 OR #51 OR #55 = **#56**

34. #17 AND #56 = **#57** **limit to Cochrane Reviews**
