# Supplementary figures and images for: Reducing risk behaviours after stroke: An overview of reviews interrogating primary study data using the Theoretical Domains Framework
Source: PLoS One. 2024 Apr 26;19(4):e0302364. doi: 10.1371/journal.pone.0302364 (PMC11051587; doi:10.1371/journal.pone.0302364)

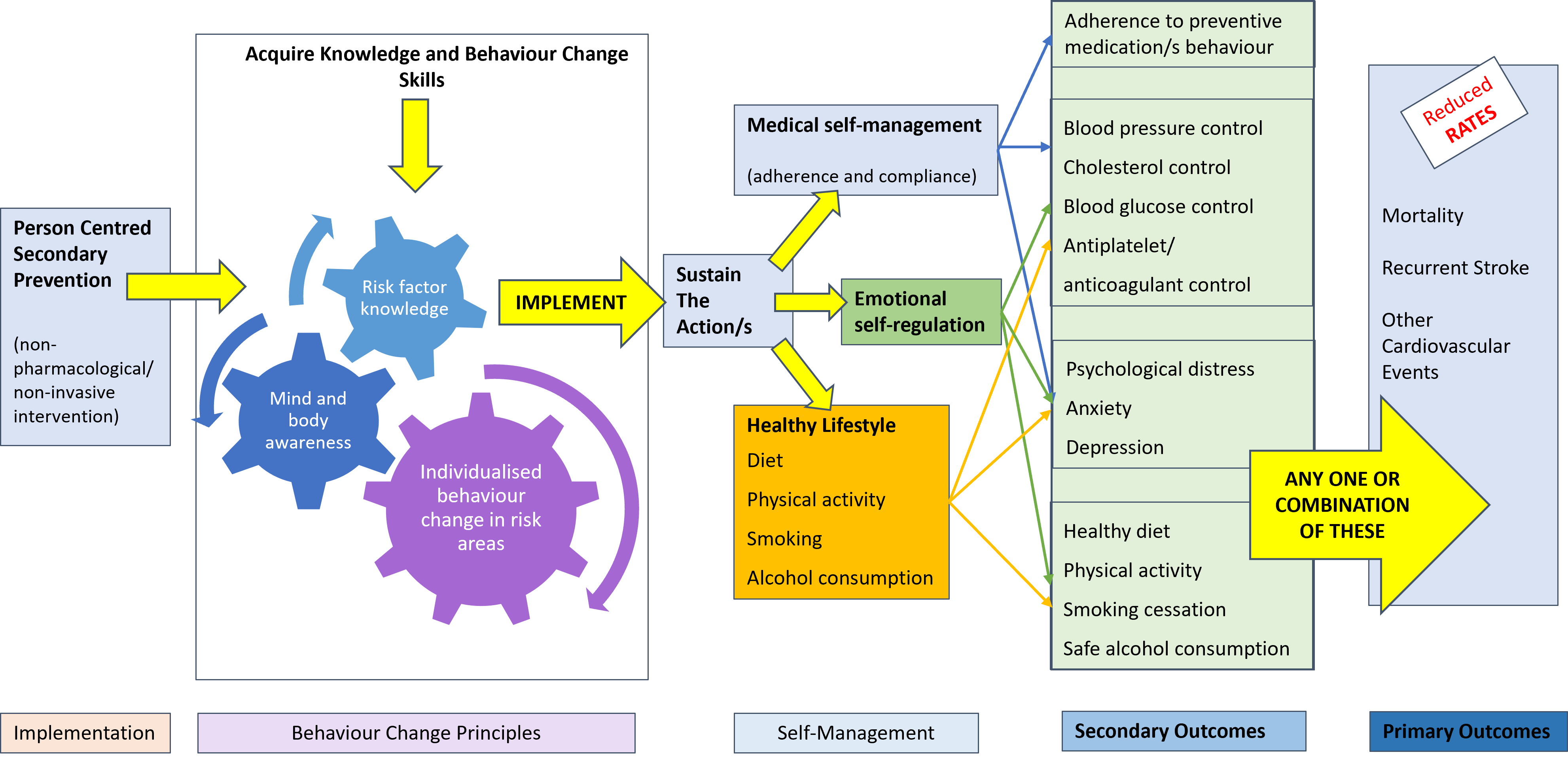

Supplement: S1 Fig — (TIF) [file pone.0302364.s006.tif]

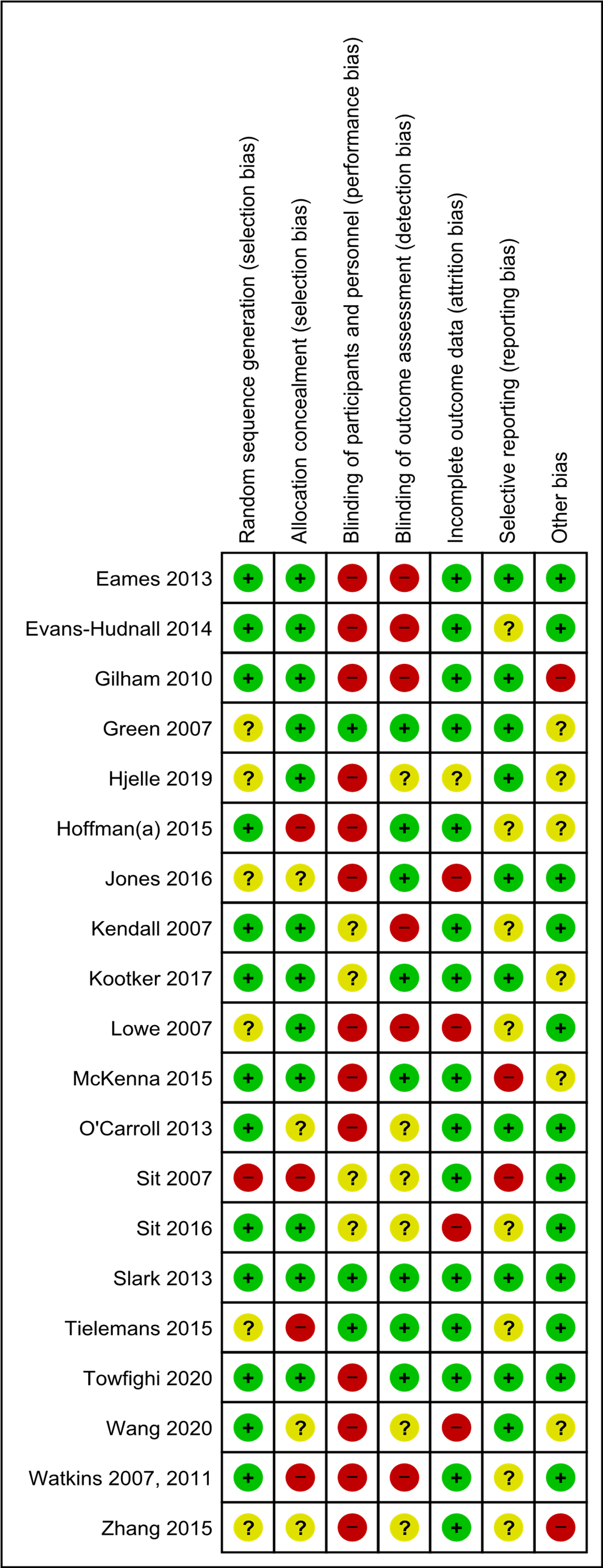

Supplement: S2 Fig — (TIF) [file pone.0302364.s007.tif]

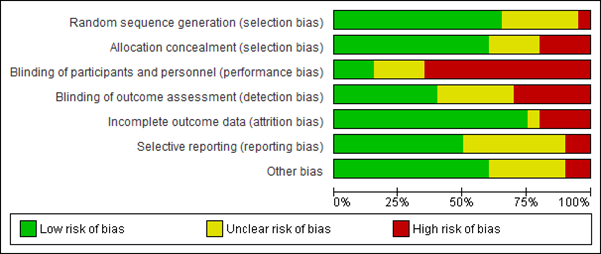

Supplement: S3 Fig — Review authors’ judgements about each risk of bias item presented as percentages across all included studies. (TIF) [file pone.0302364.s008.tif]

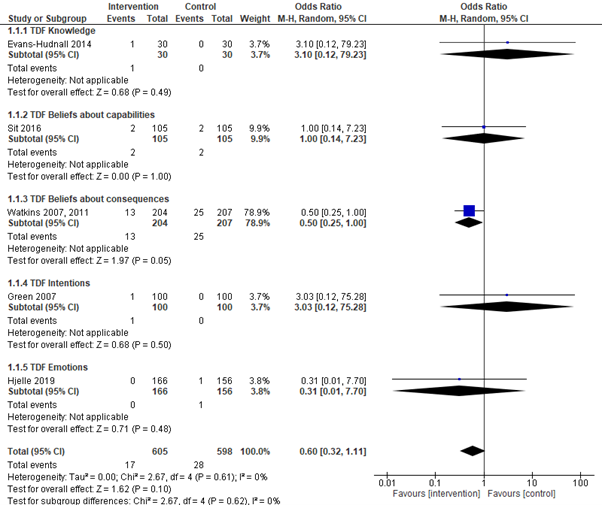

Supplement: S4 Fig — (TIF) [file pone.0302364.s009.tif]

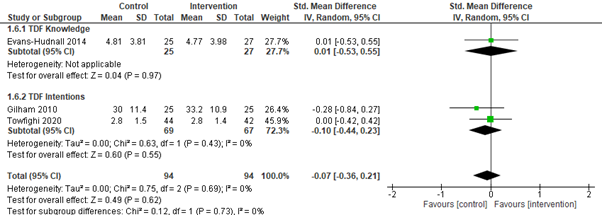

Supplement: S5 Fig — (TIF) [file pone.0302364.s010.tif]

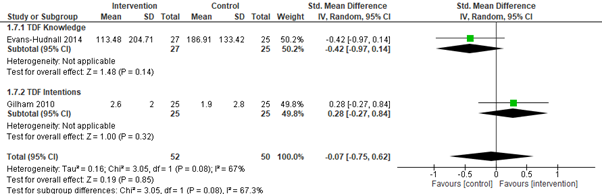

Supplement: S6 Fig — (TIF) [file pone.0302364.s011.tif]

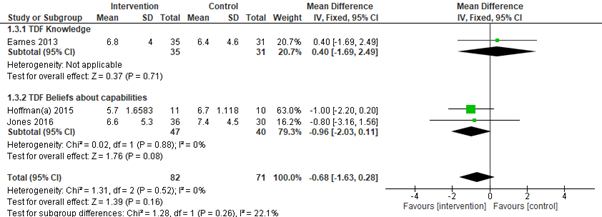

Supplement: S7 Fig — (TIF) [file pone.0302364.s012.tif]

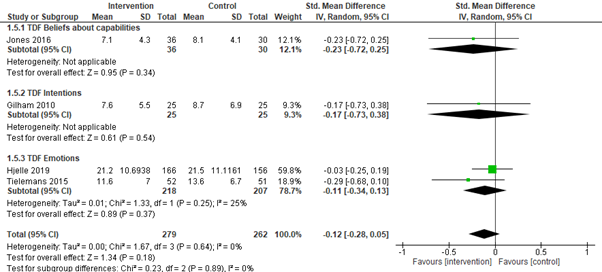

Supplement: S8 Fig — (TIF) [file pone.0302364.s013.tif]
